# Evaluation of a Cervical Stabilization Exercise Program for Pain, Disability, and Physical Impairments in University Violinists with Nonspecific Neck Pain

**DOI:** 10.3390/ijerph17155430

**Published:** 2020-07-28

**Authors:** Yi-Liang Kuo, Tsung-Han Lee, Yi-Ju Tsai

**Affiliations:** 1 Department of Physical Therapy, College of Medicine, National Cheng Kung University, Tainan 70152, Taiwan; yiliangkuo@mail.ncku.edu.tw (Y.-L.K.); jason960928@gmail.com (T.-H.L.); 2 Institute of Allied Health Sciences, College of Medicine, National Cheng Kung University, Tainan 70152, Taiwan

**Keywords:** neck pain, stabilization exercise, violinists, young adults, playing-related musculoskeletal disorders

## Abstract

Cervical stabilization exercises are frequently used to reduce pain, maximize function, and improve physical impairments for people with nonspecific neck pain. We conducted a single arm study to evaluate the effects of a home-based cervical stabilization exercise program for university violin players with nonspecific neck pain who frequently assume an asymmetrical neck posture and activate their superficial cervical flexors to stabilize the violin. Twenty violin players with nonspecific neck pain from university symphony orchestras participated in this study. All participants received assessments twice before the intervention and once immediately after a 6-week cervical stabilization exercise program. No significant differences were found between the two pretests before the intervention. After the intervention, the Numeric Rating Scale, the Neck Disability Index, the craniocervical flexion test, muscle endurance tests, cervical range of motion (all directions except flexion) tests, and cervicocephalic relocation tests (flexion and left rotation) showed improvements. The forward head posture indicated by the craniovertebral angle also slightly improved. The results of this single arm study suggest that cervical stabilization exercise is feasible and has the potential to improve physical health for collegiate violin players with nonspecific neck pain.

## 1. Introduction

Neck pain was reported as the second most frequent musculoskeletal disorder in professional violinists [1]. Playing the violin requires constant elevation of the left shoulder and lateral flexion and rotation of the cervical spine to firmly support the instrument against the chin. Prolonged exposure to an asymmetric posture and increased muscle activity in the neck–shoulder region may lead to neck pain [2,3]. The deep cervical flexors (e.g., longus capitis and colli) play a vital role in maintaining segmental stability [4,5]. Electromyography studies of patients with neck pain have reported reduced activity of the deep cervical flexors and coactivation of the superficial cervical flexors (e.g., sternocleidomastoid and anterior scalene muscles) during the craniocervical flexion test [6,7]. These impairments were also observed in violinists with neck pain [8].

Cervical stabilization exercises aiming to train deep stabilizer muscles of the cervical spine and improve coordination between superficial and deep cervical muscles have been increasingly used in recent years. These exercises require patients to control neutral spinal alignment with stabilizer muscle activations in various conditions, beginning with gentle craniocervical nods and proceeding through increased levels of extremity loading [9]. Many studies have reported positive effects of cervical stabilization exercises on the cervical range of motion, deep cervical flexors endurance, and neck pain-related disability [10,11,12,13]; however, no study has examined the effects of cervical stabilization exercises in violin players with neck pain. Violin players frequently assume an asymmetrical neck posture and activate their superficial cervical flexors to stabilize the violin for prolonged playing. Overactive superficial cervical muscles may interfere with the recruitment of deep cervical muscles in violinists with neck pain.

We aimed to evaluate the effects of a cervical stabilization exercise program for university violin players with nonspecific neck pain. We hypothesized that there would be a significant decrease in neck pain and neck disability as well as a significant increase in cervical muscle endurance and cervical range of motion after the intervention. In addition, participants would demonstrate a more ideal upper body posture (based on the plumb line definition [14]) and improved cervical proprioception.

## 2. Materials and Methods 

### 2.1. Study Design

This is a quasi-experimental study with the single-group pretest-posttest design. Participants received two pretests at week 0 and week 4 and were instructed not to receive any intervention and maintain usual activity during the baseline period. Participants started a 6-week exercise program at week 4 and had one posttest when completing the intervention at week 10. Data collection was conducted at the laboratory in the university.

### 2.2. Participants

Violin players aged 18–25 years were recruited from 2 university symphony orchestras by the investigator (THL) in person. Inclusion criteria were duration of playing the instrument longer than 5 years, practicing the violin more than 5 hours/week, constant neck pain for more than 3 months or at least 2 pain episodes in the last 3 months. Nonspecific neck pain is defined as pain perceived in the region between the superior nuchal line and the T1 spinous process and without specific underlying diseases [15]. Exclusion criteria were previous surgery in the neck and shoulder regions, current participation in a structured exercises program, current treatment for neck and shoulder pain, neurological symptoms of the upper extremities during screening tests (e.g., Spurling test and upper limb tension tests), or red flags suggesting of cancer, infection, vascular insufficiency, and etc. This study was approved by the investigators’ institutional review board (A-ER-107-073) and registered with ClinicalTrials.gov NCT04051593. Written informed consent was obtained from all participants. 

Minimum sample size was estimated based on the minimal clinically important difference (MCID) for neck pain on the Numeric Rating Scale (NRS) reported in the literature [16]. The required sample size for power of 0.8, a two-tailed α of 0.05 and an effect size of 0.68 for two dependent means was 19. We enrolled 24 participants to allow for 20% dropout in this study. For a priori power analysis, we used the G*power 3.1 software (Heinrich-Heine-Universität Düsseldorf, Germany) [17] to determine the sample size for this study.

### 2.3. Outcome Measures

The level of neck pain and disability was assessed using the NRS and neck disability index (NDI), respectively. Both are commonly used outcome measures for patients with neck pain, and considered to be reliable, valid and responsive [16]. The MCID for the NRS and NDI is 1.3 and 19%, respectively [16].

Physical impairments of the cervical spine were assessed using the craniocervical flexion test, cervical muscle endurance tests, cervical range of motion, and cervicocephalic relocation test. In addition, upper body posture was measured. Three trials were collected and averaged for each test.

**Figure 1:**
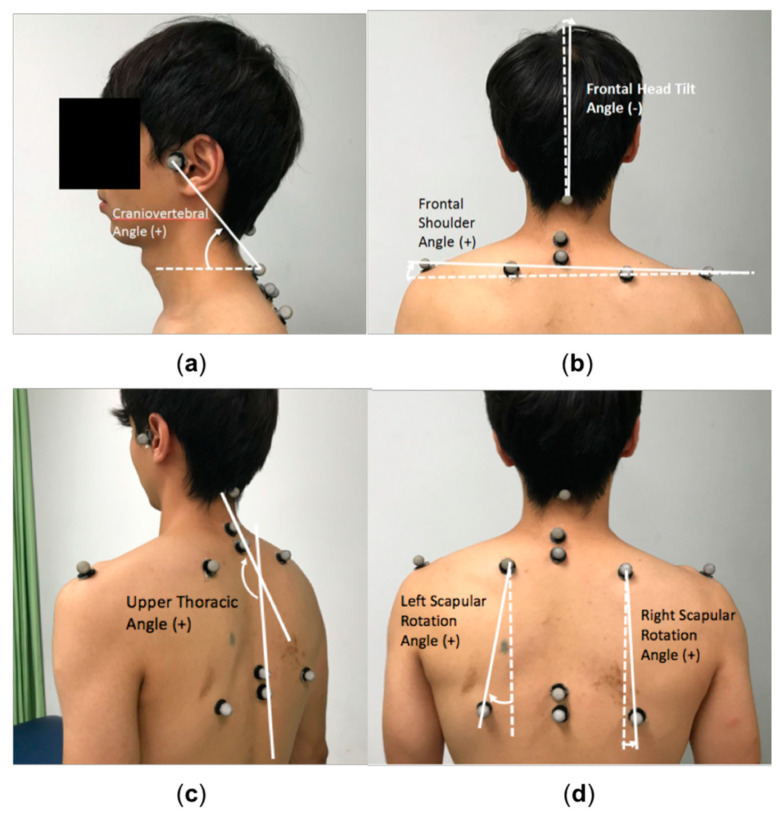
Marker placement and angle definition. (**a**) Craniovertebral angle was defined as the angle between the line connecting the tragus of the ear and the spinous process of the C7 and the horizontal line; (**b**) Frontal head tilt angle was defined as the angle between the midline of the head relative to the vertical line. Frontal shoulder angle was defined as the angle between the line connecting the left acromion and right acromion and the horizontal line; (**c**) Upper thoracic angle was defined as the angle between the line connecting the spinous processes of the T1 and T2 and the line connecting the spinous processes of the T6 and T7; (**d**) Scapular rotation angles were defined as the angle between the line connecting the superior and inferior scapular angles and the vertical line.

- Craniocervical flexion test: The craniocervical flexion test was used to assess the neuromuscular control of deep cervical flexors [18]. Participants performed a gentle head nodding action and increased the pressure reading of the pressure biofeedback unit (Stabilizer, Chattanooga Group, Dallas, TX) in 5 test increments (22, 24, 26, 28, and 30 mmHg) in a hook-lying position. Each test increment was held for 10 s with 10 s of rest between increments, and the highest achievable pressure was recorded. The craniocervical flexion test has been shown with good construct validity and moderate to good intra-rater reliability, and its MCID is 2 mmHg [18,19].
- Cervical muscle endurance tests: Cervical muscle endurance tests were measured using a stopwatch for the successful time holding the tested position [19,20]. The cervical flexors endurance test was performed in a hook-lying position by lifting the head and neck approximately 2.5 cm off the plinth while maintaining the chin tucked. The cervical extensors endurance test was performed in a prone position with the head protruding from the plinth while retracting the chin and holding the head horizontally. The minimal detectable change (MDC) for the cervical flexors endurance test is 17.8 sec [20], and the MCID for the cervical extensors endurance test is 73 sec [19].
- Cervical range of motion: Cervical range of motion was examined using a validated cervical range-of-motion device (Performance Attainment Associates, Lindstrom, MN) [21]. Participants moved their heads from their own natural neutral position into maximal flexion, extension, left lateral flexion, right lateral flexion, left rotation, and right rotation positions. The MDC for cervical range of motion test is 9.6° for flexion, 7° for extension, 9.1° for left lateral flexion, 5.9° for right lateral flexion, 6.7° for left rotation, and 7.6° for right rotation [21].
- Cervicocephalic relocation test: The cervicocephalic relocation test was used to examine cervical joint position sense [22]. Participants were blindfolded and wore a laser pointer on their heads. Participants memorized the neutral head position, performed maximal cervical movements (flexion, extension, left rotation, and right rotation), and then relocated the head to the reference position. The cervical joint reposition error was measured as the distance between the final position of the light beam of the laser pointer and the initial reference position. The MDC for the cervicocephalic relocation test is 0.8–1.0 cm for cervical flexion, 0.8–0.9 cm for cervical extension, 0.6–0.8 cm for left rotation, and 0.6–1.0 cm for right rotation [23].
- Upper body posture: Upper body posture was measured using the photographic method [24,25]. A digital camera (Canon 450D) was set on a tripod, positioned 2.5 m from the participant and 1 m above the ground. Before photography, the researcher placed reflective markers on anatomic landmarks (Figure 1). One back- and 2 lateral-view photographs of the habitual standing posture were taken and analyzed using ImageJ software (National Institutes of Health, Bethesda, MD). Five postural angles were analyzed: frontal head tilt angle, frontal shoulder angle, right and left scapular rotation angles, craniovertebral angle, and upper thoracic angle (Figure 1).

### 2.4. Interventions

The investigator (THL) instructed the participants how to perform each exercise face-to-face when the participants came to the laboratory for pretest measurements at the second time point. Then, the participants followed instructional videos and performed the exercise program at home for 20 min/day, 3 days/week, for 6 weeks. The investigator periodically contacted the participants by phone or video calls to check correctness and adherence of exercises. The exercise program progressed by increasing the repetition of the exercise during the first 3 weeks (10 reps to 15 reps to 20 reps). During the final 3 weeks, the participants changed to an elastic band with greater resistance and followed the same repetition progression during the first 3 weeks. Participants maintained an exercise log recording the date and repetitions of their exercises.

**Figure 2:**
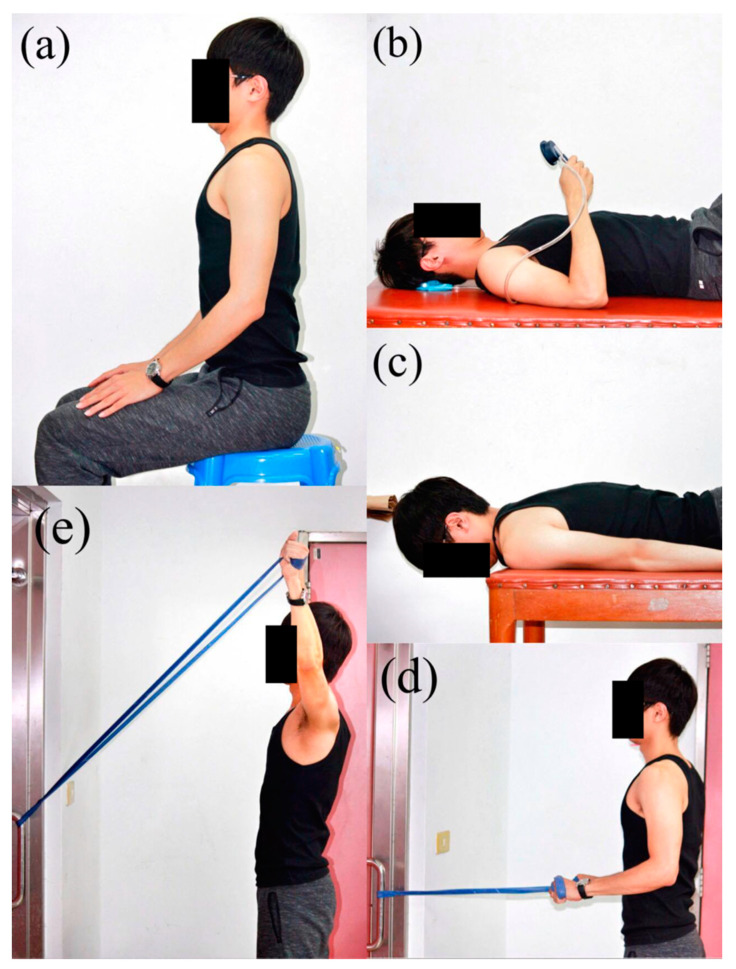
Six-week video-based exercises: (**a**) axial elongation exercise, (**b**) craniocervical flexion exercise, (**c**) craniocervical extension exercise, (**d**) rowing exercise, (**e**) Y exercise.

The exercise program consisted of axial elongation exercise, craniocervical flexion exercise, cervical extension exercise, and cervico-scapulothoracic strengthening exercises (Figure 2). Axial elongation exercise was performed to correct posture. The participants gently performed chin-in and shoulder retraction while seated and then elongated the entire spine by imaging a string pulling from the top of the head. Craniocervical flexion and cervical extension exercises were performed to retrain the deep cervical flexors and improve postural awareness. The participants were requested to learn the correct craniocervical flexion in a supine position by using a pressure biofeedback unit in the laboratory before they performed the exercise at home without pressure biofeedback. For the cervical extension exercise, the participants firstly maintained craniocervical flexion and then lifted and held the head and neck in a prone position. A rowing exercise with elastic band was performed in a seated position to strengthen shoulder extensors and scapular retractors. Y exercise with an elastic band was performed in a standing position to strengthen the lower trapezius muscles. The participants were instructed to maintain chin-in posture and spinal alignment while performing these exercises.

### 2.5. Statistical Analysis

All data were analyzed using SPSS 17.0 (SPSS Inc., Chicago, IL). The normality assumption was tested using the Shapiro–Wilk test. Descriptive statistics were summarized as means and standard deviations for normally distributed data and medians and interquartile ranges for skew data. Intraclass correlation coefficients were calculated to examine absolute agreement between the 2 pretest measurements. One-way repeated-measures analysis of variance was used to identify any statistically significant differences among the 3 time points for each dependent variable. If a significant main effect of time was identified, repeated contrasts were used to examine the difference during the baseline period and during the intervention period. Given that the NRS, NDI, and craniocervical flexion test data were not normally distributed, Friedman tests were used. Effect sizes (Pearson r) for the Wilcoxon signed-rank test and F-ratios of the repeated contrasts [26] were calculated and reported. Effect sizes are classified as small (r = 0.2), medium (r = 0.3), and large (r ≥ 0.5) [27]. The significance level was set at *p* < 0.05.

## 3. Results

Twenty-four participants were initially enrolled; however, 4 dropped out because of acute injury unrelated to the intervention and other personal problems. Twenty participants (10 men, 10 women; age: 21.2 ± 3.2 y; height: 167.8 ± 9.3 cm; weight: 57.9 ± 9.2 kg; body mass index: 20.5 ± 2.0 kg/m^2^) completed the intervention and measurements. The participants ordinarily practiced for an average of 20.1 (±7.1) hours/week and had played their instrument on average for 11.9 (± 3.8) years. The average adherence rate of the intervention calculated from the completed sessions over the total 18 sessions for exercise was 90.1%. In addition, the participants reported that they could completed the exercise program at home with little difficulty and within acceptable time.

Participants reported mild to moderate neck pain (NRS: range 3–7 points) and none to mild disability (NDI: range 4%–18%) before the intervention. No statistically significant difference was observed in the NRS and NDI during the baseline period (all *p* > 0.05, Table 1). After the intervention, the median NRS score and NDI percentage point significantly decreased by 4 and 4, respectively (*p* < 0.01, Table 1).

No statistically significant difference was observed for the craniocervical flexion, cervical muscle endurance, cervical range of motion, and cervical joint reposition tests in all directions during the baseline period (all *p* > 0.05, Table 1). After the intervention, a significant improvement was observed for the flexion, cervical muscle endurance, cervical range of motion, and cervical joint reposition tests (all *p* < 0.05, Table 1). The median pressure level achieved during the craniocervical flexion test increased by 4 mmHg. The average holding time increased by 5.4 s for the cervical flexor muscle endurance test and 78.3 s for the cervical extensor muscle endurance test. In addition, the average range of cervical extension, bilateral lateral flexion, and rotation motion significantly increased by more than 5°, and the average cervical joint reposition error on return from flexion and left rotation significantly decreased by approximately 2 cm.

The mean craniovertebral angle remained relatively unchanged during the baseline period (*p* = 0.76); however, it significantly increased by 1.8° during the intervention period (*p* < 0.01). The frontal shoulder angle slightly decreased by 0.006° during the baseline period (*p* = 0.02) and increased by 0.3° during the intervention period (*p* = 0.11). Considering the main effect of time for the frontal shoulder angle was not significant (F = 2.785, *p* = 0.11), the statistically significant planned contrast during the baseline period could be ignored. No statistically significant difference was observed in the other postural angles during the baseline or intervention periods, including the cervical lateral flexion, bilateral scapular rotation, and upper thoracic kyphosis angles (all *p* > 0.05, Table 1).

## 4. Discussion

The present study is the first to investigate the effects of cervical stabilization exercises in university violin players with nonspecific neck pain. The training format of the initial one-on-one supervision and follow-up instructional videos was acceptable and feasible, and the adherence to the home-based exercise program was high. Significant improvements were observed in neck pain, control of deep cervical flexors, and some outcome measures after the 6-week intervention. Considering that no differences in these outcome measures were logged during the baseline period, and that the two pretest measurements were moderately to highly agreeable, the differences observed after the intervention may imply the beneficial role of cervical stabilization exercises in this population. 

Our results of cervical stabilization exercise on alleviating neck pain are consistent with the results of previous studies [11,12,28]. Cervical stabilization exercise probably induces local afferent input into the central nervous system to modulate pain perception [12,29]. The exercise program conducted by Falla et al. [28] was personally instructed and supervised by an experienced physical therapist once per week for 6 weeks. The participants were also instructed to perform the exercises twice daily during the intervention period. Griffith et al. [11] executed a similar exercise program. The participants performed the exercise 5-10 times a day, more frequently than those participants in the study of Falla et al. [28]. Compared with previous studies, our participants received only one face-to-face instruction and then followed the instructional videos to practice cervical stabilization exercises thrice weekly. Previous studies have shown that multimedia-based exercise instructions can improve understanding and adherence to exercises [30,31]. Considering our positive results, instructional videos may be an effective and cost-efficient method for delivering cervical stabilization exercises. However, it is worth knowing that the difference (4%) found in the NDI during the intervention period was smaller than the previously reported MCID (19%). 

Although several studies have utilized cervical stabilization exercise [10,11,12,13,29], few studies have included an objective measurement for deep cervical flexors [13,29]. In this study, participants demonstrated a significant improvement in the craniocervical flexion test during the intervention period. The mean pressure increase (4 mmHg) was similar to that reported by a study (3.1 mmHg) [29], and both exceeded the MCID (2 mmHg) observed in people with neck pain [19]. The craniocervical flexion test was verified as a valid and reliable clinical test for deep cervical flexors [18]. Therefore, the finding may indicate that cervical stabilization exercise counteracted augmented activation of superficial cervical flexors and produced clinically meaningful improvements in deep cervical flexors. Further electromyography study is needed to confirm this inference.

In this study, cervical muscle endurance, cervical range of motion (all directions except flexion), and joint position sense for cervical flexion and left rotation showed statistically significant improvement after the intervention. The statistically significant difference (78.3 sec) found in the cervical extensor endurance test was larger than the previously reported MCID (73 sec), suggesting meaningful improvement. Compared with the previously reported MDC [21,23], the differences found in cervical extension, left and right rotation ranges as well as joint position sense for cervical flexion and let rotation after the intervention were large enough and less likely to occur by chance. Better cervical muscle endurance and joint position sense may enhance the ability to maintain spinal stability and to tolerate external mechanical loading during long duration violin playing, which might contribute to pain reduction.

Few studies have examined the effects of cervical stabilization exercise on postural correction. The craniovertebral angle is commonly used to quantify forward head posture in the literature [25,32]. The finding of a significantly increased craniovertebral angle after the intervention suggests improvement in the forward head posture. Forward head posture is often observed in people with neck pain [25,32]. Reducing forward head posture can reduce mechanical loading in postural muscles and alleviate existing neck pain [33].

This study had several limitations. Although a double pretest was used to minimize the bias caused by the one-group pretest–posttest design, the lack of a comparison group limits the inference of causality. Moreover, the participants were amateur violin players from university symphony orchestras and had low NDI scores. Their pain intensity, playing experience, and training intensity might be different from university students majoring in violin and professional violinists. Researchers should be cautious about generalizing the current findings to the other violinist populations with more severe disability. In addition, the outcome measures were performed immediately after the 6-week exercise program. The effect of cervical stabilization exercise in university violin players with neck pain must be further confirmed in a randomized controlled trial with a larger sample and follow-up tests.

## 5. Conclusions

Our study provided preliminary evidence about the effects of a cervical stabilization exercise program for university violin players with nonspecific neck pain. Compared with the baseline period, significant changes were noted in neck pain, control of deep cervical flexors, and some measures of physical impairment during the intervention period.

## Figures and Tables

**Table 1:** Descriptive and inferential statistics of outcome measures (N = 20).

Variable	Pre-Test 1	Pre-Test 2	Post-Test	Baseline Period				Intervention Period		
				ICC	Δ	*p*	r	Δ	*p*	r
Numeric rating scale (point)	4 (0)	4 (1)	0 (2)	0.953	0	0.08	−0.39 ^1^	4	< 0.01	−0.89 ^1^
Neck disability index (%)	8 (6)	8 (7.5)	4 (6)	0.958	0	0.32	−0.22 ^1^	4	< 0.01	−0.88 ^1^
Craniocervical flexion test (mmHg)	20 (2)	20 (2)	24 (4)	0.674	0	> 0.99	0 ^1^	−4	< 0.01	−0.77 ^1^
Cervical muscles endurance (sec)										
Flexors	9.2 ± 3.4	9.1 ± 3.0	14.5 ± 4.8	0.919	0.1	0.80	0.06	−5.4	< 0.01	0.83
Extensors	72.0 ± 63.6	76.9 ± 58.6	155.2 ± 75.3	0.979	−4.9	0.22	0.28	−78.3	< 0.01	0.70
Cervical range of motion (degree)										
Flexion	42.7 ± 15.9	44.0 ± 14.8	48.4 ± 12.0	0.777	−1.4	0.65	0.10	−4.4	0.08	0.40
Extension	52.7 ± 12.0	50.7 ± 17.3	59.6 ± 14.3	0.822	1.9	0.47	0.17	−8.9	0.01	0.57
Left SB	36.0 ± 9.8	34.1 ± 8.8	40.2 ± 8.6	0.874	1.9	0.18	0.31	−6.1	< 0.01	0.72
Right SB	36.5 ± 8.1	34.8 ± 10.7	40.2 ± 9.6	0.887	1.7	0.22	0.28	−5.4	< 0.01	0.60
Left rotation	57.1 ± 10.0	54.1 ± 12.5	66.9 ± 10.7	0.705	3.0	0.23	0.27	−12.8	< 0.01	0.77
Right rotation	56.5 ± 14.5	55.1 ± 12.4	65.8 ± 14.0	0.853	1.4	0.55	0.14	−10.6	< 0.01	0.71
Cervical joint position sense (cm)										
Flexion	6.3 ± 2.3	6.7 ± 3.1	4.1 ± 1.8	0.818	−0.5	0.35	0.22	2.6	< 0.01	0.61
Extension	5.7 ± 2.7	5.8 ± 3.7	4.6 ± 1.6	0.873	−0.1	0.84	0.05	1.3	0.09	0.38
Left rotation	5.3 ± 1.9	6.1 ± 2.7	4.5 ± 2.1	0.381	−0.8	0.22	0.28	1.6	< 0.01	0.57
Right rotation	8.0 ± 3.3	7.4 ± 3.3	5.7 ± 3.0	0.842	0.6	0.26	0.26	1.6	0.05	0.43
Upper body posture (degree)										
Frontal head tilt angle ^2^	−1.2 ± 8.1	−1.2 ± 8.1	−0.6 ± 2.5	1.000	0.002	0.52	0.15	−0.6	0.72	0.08
Frontal shoulder angle	0.4 ± 1.7	0.4 ± 1.7	0.02 ± 1.3	1.000	−0.006	0.02	0.51	0.3	0.11	0.36
Scapular rotation angle ^3^ (right)	2.1 ± 9.5	2.1 ± 9.6	−0.1 ± 3.9	1.000	−0.003	0.61	0.12	2.2	0.18	0.31
Scapular rotation angle ^3^ (left)	3.7 ± 8.7	3.7 ± 8.7	0.3 ± 3.5	1.000	0.004	0.31	0.23	3.4	0.06	0.42
Craniovertebral angle	51.4 ± 5.4	51.4 ± 5.4	53.2 ± 5.0	1.000	0.002	0.76	0.07	−1.8	< 0.01	0.64
Upper thoracic angle	149.9 ± 13.4	149.5 ± 13.4	149.6 ± 10.1	1.000	−0.04	0.37	0.21	−0.08	0.96	0.01

Values are expressed as mean ± standard deviations or median (interquartile range). ICC_3,k_ = intraclass correlation coefficients, Δ = mean difference, r = effect size. ^1^ The denoted effect sizes are calculated from the Wilcoxon signed-rank test, and the other undenoted effect sizes are calculated from the F-ratios of the repeated contrasts. ^2^ Positive value indicates left side bending of the head, and negative value indicates right side bending of the head. ^3^ Positive value indicates scapular upward rotation, and negative value indicates scapular downward rotation.
